# Orthogonal Space-Time Block Coding for Double Scattering V2V Links with LOS and Ground Reflections

**DOI:** 10.3390/s23239594

**Published:** 2023-12-03

**Authors:** Miguel Gutiérrez Gaitán, Gowhar Javanmardi, Ramiro Sámano-Robles

**Affiliations:** 1 Facultad de Ingeniería, Universidad Andres Bello, Santiago 8370146, Chile; miguel.gutierrez@unab.cl; 2 CISTER/ISEP, Polytechnic Institute of Porto, 4200-465 Porto, Portugal; gowha@isep.ipp.pt; 3 Faculty of Engineering, University of Porto, 4099-002 Porto, Portugal

**Keywords:** LOS, MIMO, STBC, two-ray model, V2V

## Abstract

This work presents the performance analysis of space-time block codes (STBCs) for vehicle-to-vehicle (V2V) fast-fading channels in scenarios with modified line-of-sight (LOS). The objective is to investigate how the V2V MIMO (multiple-input multiple-output) system performance is influenced by two important impairments: deterministic ground reflections and an increased Doppler frequency (time-variant channels). STBCs of various coding rates (using an approximation model) are evaluated by assuming antenna elements distributed over the surface of two contiguous vehicles. A multi-ray model is used to study the multiple constructive/destructive interference patterns of the transmitted/received signals by all pairs of Tx–Rx antenna links considering ground reflections. A double scattering model is used to include the effects of stochastic channel components that depend on the Doppler frequency. The results show that STBCs are capable of counteracting fades produced by destructive self-interference components across a range of inter-vehicle distances and for a range of Doppler frequency values. Notably, the effectiveness of STBCs in deep fades is shown to outperform schemes with exclusive receive diversity, despite the interference created by the loss of orthogonality in time-varying channels with a moderate increase of Doppler frequency (mainly due to higher vehicle speeds, higher frequency or shorter time slots). Higher-order STBCs with rate losses are also evaluated using an approximation model, showing interesting gains even for low coding rate performance, particularly when accompanied by a multiple antenna receiver. Overall, these results can shed light on how to exploit transmit diversity in time-varying vehicular channels with modified LOS.

## 1. Introduction

Vehicle-to-vehicle (V2V) communication is a promising yet challenging aspect of vehicular networks and future intelligent transportation systems (ITS) [1]. V2V communication performance is crucial for several safety-critical vehicular applications, (e.g., platooning [2] or cooperative adaptive cruise control (CACC) [3], which rely on advanced vehicle networking capabilities to improve transportation efficiency and road safety).

Multiple-input multiple-output (MIMO) systems are one of the prospective upgrades to be implemented in future connected vehicles [4] (including platoons and CACC), with the aim of improving channel capacity and reliability with minimum spectrum efficiency losses. MIMO theory and its applications are, in fact, a well-known subject in wireless communications [5], offering vast practical and theoretical knowledge with proven technological success.

Specifically, space-time block codes (STBCs) constitute a classical approach to achieve transmit diversity in MIMO systems. In particular, the pioneering work of Alamouti in [6] proposed an effective and low-complexity pre-coding/decoding scheme using resources in time (time slots) and space (antennas) that paved the way for several other coding techniques that achieved efficient diversity at the transmitter side, notably obtaining performance results that were equivalent to a maximum-ratio combining (MRC) receiver [7].

While the advantages of STBCs in conventional stochastic fading channels are relatively well understood, their potential to counteract fading due to the combined effect of deterministic ground reflections and fast-fading (time-varying) channels has not been fully explored for vehicular applications, a challenge we attempt to address in this paper.

Based on the theory of the two-ray model, ground reflections introduce interference patterns mainly when the angle of reflection is lower than a critical value. At this critical point, the phenomena of total reflection creates a deep fade. In the region of distances or angles beyond the main deep fade, the path-loss exponent changes creating a relative absence of fades. This means that ground reflections will have their main impact in scenarios with angles below the critical point, which in turn means with reduced inter-vehicular distance and/or increased height of the antennas.

Channel time variability is commonly introduced in wireless networks as a function of the Doppler frequency. The Doppler frequency is directly connected to an increase in the speed of the vehicle but also to the increase of operational frequency and/or reduction of transmission periods. Therefore, in practical scenarios, we expect to have deterministic ground reflections and higher Doppler for shorter distances between vehicles, increased vehicle speeds, and also in environments dominated by scattering, such as in urban and dense urban scenarios.

In this article, we investigate the performance of STBCs in V2V MIMO links scenarios considering the combined effect of ground reflections and fast-fading vehicular channels (see e.g., [8,9] for further motivations). More concretely, this work is an extension of the conference paper in [10], which focused exclusively on deterministic V2V channels with ground reflections. This means our previous work ignored stochastic scattering. In this work, to make the channel more realistic, we introduce a stochastic component with a double scattering distribution that is typical in V2V geometrical-based stochastic models. The end result is a V2V MIMO time-variant channel model with double fading and a LOS deterministic component that explicitly considers ground reflections.

The deterministic analysis is built upon our prior work in [11], where we first established a V2V channel model to account for those self-interference patterns that appear when using multiple antennas in the presence of specular ground reflections. The stochastic model is replicated from another work [12] that accounts for a set of distributed scatterers around each vehicle, with the signal model being the result of a double interaction between these two sets of scatterers.

More importantly, the main results here presented indicate that under these channel conditions, time variability introduces a self-interference term that destroys the orthogonality of the codes, thus reducing performance. The objective of our study is thus to evaluate to what extent performance is reduced due to this impairment and if communication can still occur under these circumstances.

To verify the above inquiry, STBCs of various coding rates are evaluated across a range of inter-vehicle distances for different assumptions of Rice factor and Doppler shifts. The results show that for moderate vehicle speeds and for high values of the Rice factor, the performance of STBCs still remains a good option for vehicular communications despite the loss of orthogonality. However, the achieved performance can be rapidly degraded once the optimum range of conditions changes.

The organization of this paper is as follows. Section 2 provides some related works and motivation. Section 3 presents the channel model containing deterministic and stochastic components. This means it presents the evaluation of the combined effects of ground reflections and high Doppler freqeuncies. Section 4 introduces the generic MIMO signal model, including the example of the conventional Alamouti scheme for 2 × 1 MIMO systems for links with double fading, a conventional LOS component, and time-variant channels. Section 5 presents the modeling assumption for high-performance metrics of general STBCs of different order. Then, Section 6 discusses the results of simulation and distributions for a number of scenarios considering the previous results for LOS channel models (i.e., no scattering) and statistics of signal-to-noise-plus-interference (SINR) ratio that arise in time-variant channels (scattering + LOS). Finally, Section 7 provides conclusions.

## 2. Related Work and Motivation

MIMO technology lies at the core of improved connectivity for the next generation of wireless communication systems [13]. STBCs are simple but rather powerful MIMO solutions with still unexplored performance in vehicular applications. A number of works justify the use of MIMO using deterministic LOS channels for V2V applications [8,9,14,15]. In this context, our previous work in [10] explored the impact of ground reflections on the deterministic fading distribution of the MIMO V2V channel. To the best of our knowledge, this has been addressed partially in [16], but without considering its impact on capacity performance.

However, there are practically no experimental or theoretical approaches to study how these two issues that can appear simultaneously in vehicular networks can affect performance: *ground reflections* and *increased Doppler frequency*. Accordingly, our work points towards this direction by proposing a hybrid model with deterministic ground ray reflections and a stochastic scattering model that induces time variations and fading in the channel distribution.

To summarize, we consider the main contributions of this paper (as the extension of the conference paper in [11]) to be the following:

1. To investigate the performance of STBC codes for V2V MIMO when the time-variant channel component affects the orthogonality of the codes;
2. To provide a model for the interaction of two-channel components: the deterministic (a modified LOS with multiple ground reflections) and the stochastic component (a model based on rings of scatterers around both vehicles).

*Notation*. Scalar variables are denoted by lowercase letters. The variable *i* represents the imaginary number $i=\sqrt{-1}$, E[·] is the statistical average operator, and $\mathrm{CN}(\mu ,\sigma^{2})$ denotes a complex circular Gaussian distribution with mean μ and variance $\sigma^{2}$. Vector and matrix variables are denoted, respectively, by bold lowercase and capital letters. ${\left(\cdot \right)}^{T}$ and ${\left(\cdot \right)}^{H}$ denote, respectively, the conventional vector and the Hermitian vector transpose operators. The probability density (PDF) and cumulative density function (CDF) of a random variable *X* are denoted, respectively, by $f_{X}\left(x\right)$ and $F_{X}\left(x\right)=\int_{-\infty }^{x}f_{X}\left(u\right)du$.

## 3. V2V Channel Model

**Figure 1:**
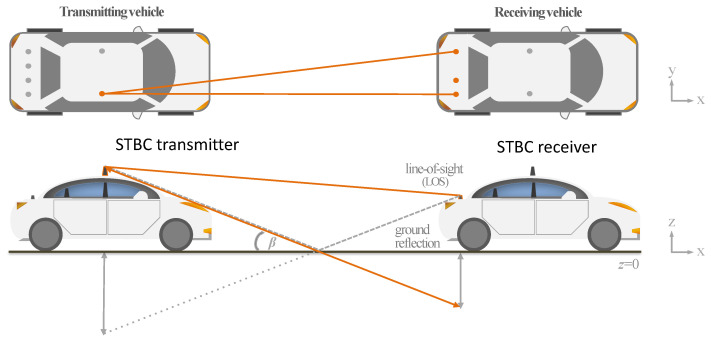
V2V LOS link showing: (**above**) an aerial view of the V2V LOS channel for STBC communication and (**bottom**) the geometrical basis of the two-ray model with both the LOS and ground reflected paths.

Consider the V2V scenario depicted in Figure 1 where each vehicle contains two horizontal uniform linear arrays of *N* antennas each. The first array is placed at the rooftop of each vehicle, while the other is at the corresponding front (or rear) part (bumper) of the vehicle. The arrays are thus mirrored in the contiguous vehicle, with $2N=N_{Tx}=N_{Rx}$ antennas by car, where $N_{Tx}$ and $N_{Rx}$ are, respectively, the total number of transmit and receive antennas. The configuration can be better understood by looking at Figure 1, where one array of 4 antennas is located at the rooftop of each vehicle, and a second array is located in the front or rear bumper of the vehicle, depending on whether communication takes place with the vehicle in front or behind. Note that all arrays contain the same number of elements *N*. The channel between any of the *j*th and *k*th antenna elements at time *t* is denoted as $h_{j,k}\left(t\right)$ and is defined as the contribution of the deterministic and the stochastic component (i.e., $h_{j,k}\left(t\right)=h_{j,k}^{d}+h_{j,k}^{s}\left(t\right)$). The deterministic component contains the original LOS (line-of-sight) component and also all the deterministic ground reflections.

### 3.1. Deterministic V2V Channel Component (LOS+Ground Reflections)

Let us assume that $h_{j,k}^{d}$ is described by the classical two-ray model. Note that in the related literature, this propagation model has been recognized as a good predictor of the received signal strength in V2V deterministic links [17,18]. Formally, we consider this channel component to be described as in [11]:

(1) $$h_{j,k}^{d}=\sqrt{P_{T}G_{T}G_{R}/4\pi N_{Tx}}\left(e^{2\pi i{\tilde{d}}_{j,k}}/{\tilde{d}}_{j,k}+\rho j,ke^{2\pi i{\tilde{d}}_{j,k}^{\left(gr\right)}}/{\tilde{d}}_{j,k}^{\left(gr\right)}\right)$$

where ${\tilde{d}}_{j,k}=d_{j,k}/\lambda$ and ${\tilde{d}}_{j,k}^{\left(gr\right)}=d_{j,k}^{\left(gr\right)}/\lambda$, are, respectively, the direct and ground reflected electric distances computed as a function of $d_{j,k}$ and $d_{j,k}^{\left(gr\right)}$, the distance of the respective LOS and ground reflected path. $P_{T}$ is the average Tx power per symbol, and $G_{T}$ and $G_{R}$ are the respective Tx and Rx antenna gains. λ is the operational wavelength, $i=\sqrt{-1}$, and $\rho_{j,k}$ is the reflection coefficient, which can be formally expressed as follows (modification of [19]):

(2) $$\rho_{j,k}=\frac{A\sin\beta_{j,k}+B\left(\sqrt{n_{r}^{2}-\cos{\beta_{j,k}}^{2}}+in_{i}\right)}{n_{r}^{2}\sin\beta_{j,k}+\left(\sqrt{n_{r}^{2}-\cos{\beta_{j,k}}^{2}}+in_{i}\right)},$$

where $A=n_{r}^{2}$ and B=1 for vertical polarization, and A=1 and B=−1 for horizontal polarization. β is the angle of reflection; $n_{r}$ and $n_{i}$ denote, respectively, the real and imaginary parts of the complex refractive index of ground $n_{gr}$, given by $n_{gr}=n_{r}+in_{i}=\sqrt{\epsilon_{r}-i\frac{\sigma \lambda }{\epsilon_{0}2\pi c}}$, where *c* is speed of light; and $\epsilon_{r}$ and σ denote, respectively, the relative permittivity and conductivity of the asphalt pavement [20].

### 3.2. Stochastic V2V Channel Component

The stochastic channels will be modelled as random processes with double scatterer interaction. The model employs a sum of sinusoid signals created by a set of uniformly distributed scatterers around both of the vehicles. The difference in the angle of arrivals (AoAs) to different antenna elements defines the correlation statistics of the multiple antenna model. This can be written mathematically as follows [12]:

(3) $$h_{j,k}^{s}\left(t\right)=\sum_{u=1}^{N_{s}}\sum_{w=1}^{N_{s}}C_{u,w}e^{i\pi F_{d}\left(\cos\left(\tau_{u,j}\right)+\cos\left(\nu_{w,k}\right)\right)t+r_{u,w}},$$

where $C_{u,w}$ is a normalization factor, $F_{d}=vf/c$ is the maximum Doppler frequency, *v* is the vehicle speed, $\tau_{u,j}$ is the angle of departure (AoD) for ray *u* from antenna *j*, $\nu_{w,k}$ is the AoA of ray *w* in antenna *k*, and $r_{u,w}$ is a random phase. The Rice factor is defined as the ratio of the power in the line-of-sight component to the average power in the stochastic component: $K=\frac{E[|h^{LOS}{|}^{2}]}{E[|h^{S}{|}^{2}]}$.

This paper focuses on how an increase in Doppler frequency can affect the orthogonality of STBCS and therefore reduce their ability to counteract fades. From the previous formula of Doppler frequency, an increased value of $F_{d}$ can be obtained either by increasing the vehicle speed or the operational frequency or by reducing the transmission period. The double scattering model is an extension of the well-known Jakes’ model used in SISO (single-input single-output) systems adapted to a V2V MIMO setting. Therefore, the model is a theoretical tool we use to investigate the effects of a time-varying channel. In real life, scattering distributions may differ, and other effects might affect the statistics of double scattering interaction models.

## 4. MIMO Model

Considering a space-time block code mapping a set of $N_{Tx}$ symbols to $N_{Tx}$ transmit antennas over a set of *M* time slots, the MIMO model in the time slot *m* considering $N_{Tx}$ transmit antennas and $N_{Rx}$ receiving antennas can be defined as

(4) x(m)=H(m)s(m)+v(m),

where $s\left(m\right)={\left[s_{0}\left(m\right),s_{1}\left(m\right),\ldots ,s_{N_{Tx}-1}\left(m\right)\right]}^{T}$ is the vector of transmitted symbols across the different antennas, and v(m) the vector of a zero-mean additive circular complex Gaussian noise $v\left(m\right)∼\mathrm{CN}(0_{N_{Rx}},\sigma_{v}^{2}I_{N_{Rx}}$). $H_{N_{Rx}\times N_{Tx}}$ is the MIMO channel matrix of size $N_{R_{x}}\times N_{T_{x}}$, which corresponds to the transpose matrix formed by the elements $h_{j,k}$, x(m) is the vector of received symbols in the time slot *m*, and $0_{n}$ and $I_{n}$ denote, respectively the vector of zeros and the identity matrix of order *n*.

Given the code rate *R* defined as $R=N_{Tx}/M$, the mapping of the symbols to the antennas in different time slots can also be represented with a single matrix $S_{N_{Tx},R}$ of individual elements $s_{j,m}$, where *m* and *j* denote the respective time and Tx antenna indexes.

### Alamouti Code

The particular case of Alamouti [6] coding for a 2×1 system uses the following encoding matrix:


$$S={\left(\begin{array}{cc} s_{1} & s_{2}\\ -s_{2}^{*} & s_{1}^{*} \end{array}\right)}^{T}.$$

Considering the channel for this case as $H\left(1\right)=\left(\begin{array}{c} h_{1}\left(1\right)\;h_{2}\left(1\right) \end{array}\right)$ and $H\left(2\right)=\left(\begin{array}{c} h_{1}\left(2\right)\;h_{2}\left(2\right) \end{array}\right)$, the received signal can be written as follows:

$$r={\left[H{\left(1\right)}^{T}\;H{\left(2\right)}^{T}\right]}^{T}S+v.$$

The received signals can thus be rewritten as follows:

(5) $$r_{1}=h_{1}\left(1\right)s_{1}+h_{2}\left(1\right)s_{2}+\nu_{1},$$

(6) $$r_{2}=h_{1}\left(2\right)s_{2}^{*}-h_{2}\left(2\right)s_{1}^{*}+\nu_{2}.$$

Let us now use the combining mechanism of the Alamouti decoding process [6]:

$${\hat{s}}_{1}=h_{1}^{*}\left(1\right)r_{1}-h_{2}\left(2\right)r_{2}^{*}$$

$$=h_{1}^{*}\left(1\right)h_{1}\left(1\right)s_{1}+h_{1}^{*}\left(1\right)h_{2}\left(1\right)s_{2}-h_{2}\left(2\right)h_{1}^{*}\left(2\right)s_{2}+h_{2}\left(2\right)h_{2}^{*}\left(2\right)s_{1}+$$

$$h_{1,1}\nu_{1}-h_{2}^{*}\left(2\right)\nu_{2}^{*}.$$

We can observe that the time-variant channel introduces an interference term in the desired symbol $s_{1}$. This is the main effect that will be investigated in this paper, as well as how the time-variance of the channel can degrade the orthogonality of STBCs. The SINR (signal-to-interference-plus-noise ratio) is thus given by:

(7) $$\Gamma_{1}=\frac{|h_{1}^{*}\left(1\right)h_{1}\left(1\right)+h_{2}\left(2\right)h_{2}^{*}{\left(2\right)|}^{2}}{|h_{1}^{*}\left(1\right)h_{2}\left(1\right)-h_{2}\left(2\right)h_{1}^{*}{\left(2\right)|}^{2}+\left(\right|h_{1}{\left(1\right)|}^{2}+|h_{2}\left(2\right){|}^{2})\sigma_{v}^{2}}.$$

**Figure 2:**
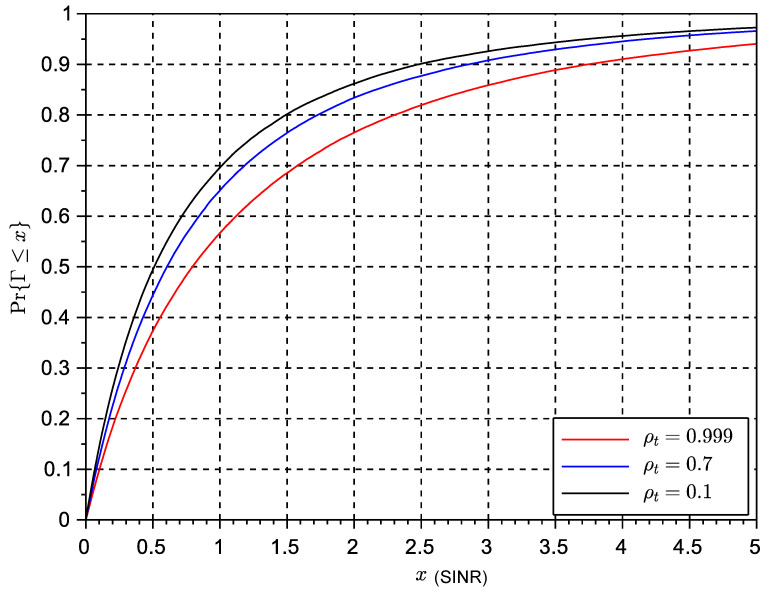
CDF of the SINR of an Alamouti space-time block code with different temporal correlation coefficients $\rho_{t}$ and a Rice factor of $K_{dB}=-30$ dB.

**Figure 3:**
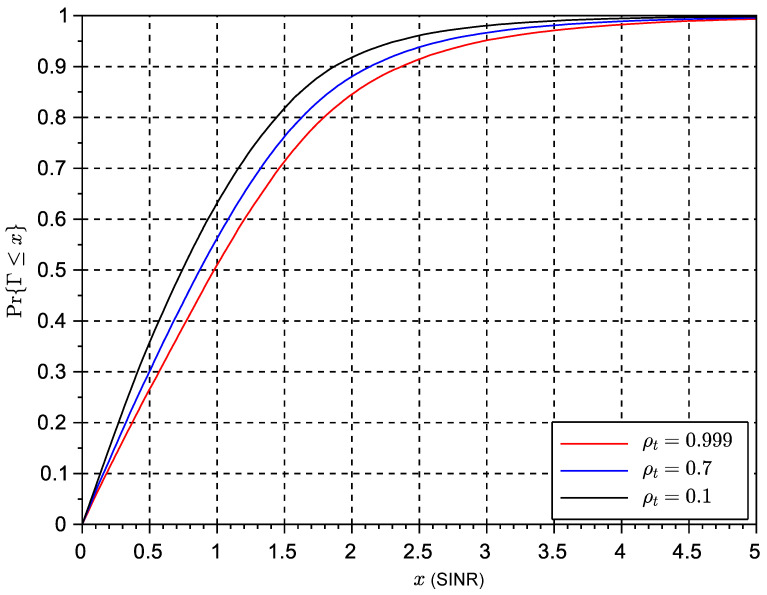
CDF of the SINR of an Alamouti space-time block code with different temporal correlation coefficients $\rho_{t}$ and a Rice factor of $K_{dB}=5$ dB.

Let us now reformulate the cross-channel dependencies. These dependencies can be approximated by the following linear correlation model: $h_{1}\left(1\right)=\mu +\phi_{1}\left(1\right)$, $h_{2}\left(2\right)=\mu +\phi_{2}\left(2\right)$, $h_{2}\left(1\right)=\mu +\psi \left(\rho_{t}\phi_{2}\left(2\right)+\sqrt{1-\rho_{t}^{2}}w_{2}\right)$ and $h_{1}\left(2\right)=\mu +\psi \left(\rho_{t}\phi_{1}\left(1\right)+\sqrt{1-\rho_{t}^{2}}w_{1}\right)$, where μ represents the deterministic component, $\rho_{t}$ represents a temporal correlation coefficient, and $w_{1}$, $w_{2}$, $\phi_{1}\left(1\right)$, $\phi_{2}\left(2\right)$ are i.i.d complex Gaussian random variables with variance γ. By substituting the above expressions in (7), the SINR expression reduces to:

(8) $$\Gamma_{1}=\frac{\left(\right|h_{1}{\left(1\right)|}^{2}+|h_{2}{\left(2\right)|}^{2}{)]}^{2}}{I+N_{o}},$$

where $I={\left[h_{1}^{*}\left(1\right)w_{1}+h_{2}^{*}\left(2\right)w_{2}+\left(\mu^{*}\phi_{1}^{*}\left(1\right)-\mu \phi_{2}\left(2\right)\right)\left(1-\rho_{t}\right)\right]}^{2}$, and $N_{o}=h_{1}^{*}\left(1\right)w_{1}+h_{2}^{*}\left(2\right)w_{2}$. Furthermore, when the channel model drops the line-of-sight component, the SINR expression becomes:

(9) $$\Gamma_{1}=\frac{|h_{1}{\left(1\right)|}^{2}+{\left|h_{2}\left(2\right)\right|}^{2}}{\tilde{g}+\sigma_{v}^{2}},$$

where $\tilde{g}$ is a double Rayleigh random variable with variance $\gamma^{2}$. An approximation of the SINR expression is proposed here where the interference term depends on the deviation between temporal components:

(10) $$\Gamma_{1}=\frac{\left(\right|h_{1}{\left(1\right)|}^{2}+|h_{2}\left(2\right){{|}^{2})}^{2}}{I+N_{o}},$$

where $I={\left(h_{1}\left(1\right)-h_{1}\left(2\right)\right)}^{2}+{\left(h_{2}\left(2\right)-h_{2}\left(1\right)\right)}^{2}$, and $N_{o}=h_{1}^{*}\left(1\right)w_{1}+h_{2}^{*}\left(2\right)w_{2}$. Figure 2 shows the CDF statistics of the Alamouti receiver considering a double Rice link with $K_{\mathrm{dB}}=-30$ dB and different values of the temporal correlation coefficient. Figure 3 shows the results using a Rice factor of 5 dB. In both cases, the x-axis is the SINR value (Γ) in linear scale. We can observe that the effects of increased Doppler tend to degrade SINR performance, but this degrading effect seems to be only evident for low values of the temporal correlation coefficient. This suggests that the Alamouti algorithm could be resilient to a moderate increase of Doppler frequency. In all results, we assume a simple line-of-sight component with random phase. In all the results, we have used double scattering statistics.

## 5. Performance Model and Metrics

The STBC receiver decodes the signals stored over the *M* time slots of the duration of the space-time block coding scheme. We will use a straightforward extension of the signal-to-interference plus noise ratio (SINR) given by the extension of the Alamouti expression in (7), which is given by:

(11) $$\Gamma =\frac{|\sum_{j}h_{j}{\left(j\right)}^{*}h_{j}\left(j\right){|}^{2}}{\sum_{j}|h_{j}{\left(j\right)|}^{2}\sigma_{v}^{2}+\left(\right|\sum_{j,k,p,q,k\neq j,p\neq q}{\left(-1\right)}^{q}h_{j}{\left(k\right)}^{*}h_{p}\left(q\right){|}^{2})}.$$

Under the approximation used for the Alamouti case in (10), we can simplify the above formula to the following expression:

(12) $$\Gamma =\frac{|\sum_{j}h_{j}{\left(j\right)}^{*}h_{j}\left(j\right){|}^{2}}{\sigma_{v}^{2}+{\left|\sum_{j}\sum_{k\neq j}h_{j}\left(j\right)-h_{j}\left(k\right)\right|}^{2}}.$$

Under the generalized temporal correlation model, *h*, the above expression reduces to

(13) $$\Gamma =\frac{\sum_{j,j}h_{j}{\left(j\right)}^{*}h_{j}\left(j\right)}{I+\sigma_{v}^{2}},$$

where *I* is a double Rayleigh random variable. The instantaneous capacity is thus given by

(14) $$C=R\log_{2}\left(1+\Gamma \right),$$

where the code rate *R* is approximated by the boundary given in [21] (i.e., $R=\frac{n_{0}}{n_{0}+1}$), and $n_{0}=2N$. The different codes will be investigated either as capacity or as the SINR metric. We recall that the SINR metric is unique to channels with fast temporal variation, and therefore in those cases it is more convenient or easier to investigate that metric. For the purposes of benchmark analysis, the average SNR of the maximum-ratio combining (MRC) and equal-gain combining (EGC) receivers are given by [11]:

(15) $$\Gamma_{MRC}=\alpha \sum_{j=1}^{N_{Rx}}{\left|\sum_{k=1}^{N_{Tx}}\left(\frac{e^{2\pi i{\tilde{d}}_{j,k}}}{{\tilde{d}}_{j,k}}+\Gamma_{j,k}\frac{e^{2\pi i{\tilde{d}}_{j,k}^{\left(gr\right)}}}{{\tilde{d}}_{j,k}^{\left(gr\right)}}+h_{j,k}^{s}\right)\right|}^{2},$$

and

(16) $$\Gamma_{EGC}=\frac{\alpha }{N_{Rx}}{\left|\sum_{k=1}^{N_{T_{x}}}\sum_{j=1}^{N_{R_{x}}}\left(\frac{e^{2\pi i{\tilde{d}}_{j,k}}}{{\tilde{d}}_{j,k}}+\Gamma_{j,k}\frac{e^{2\pi i{\tilde{d}}_{j,k}^{\left(gr\right)}}}{{\tilde{d}}_{j,k}^{\left(gr\right)}}+h_{j,k}^{s}\right)\right|}^{2},$$

respectively, where $\alpha =\frac{P_{T}G_{T}G_{R}}{N_{Tx}{\left(4\pi \right)}^{2}\sigma_{v}^{2}}$.

### Performance Metrics

Let us define the outage probability of the V2V MIMO channel as the probability that the ratio of the instantaneous capacity to the free-space loss (FSL) capacity value falls below a threshold (ξ) in the range of inter-vehicle distances $\left[d_{min},d_{max}\right]$:

(17) $$\Theta_{C}=E_{d}\left[\mathrm{Pr}\left\{C/C_{FSL}\leq \xi \right\}\right]=E_{d}\left[F_{\tilde{C}}\left(\xi \right)\right]=\frac{\int_{d_{min}}^{d_{max}}F_{\tilde{C}}\left(\xi \right)dy}{d_{max}-d_{min}},$$

where *y* is the auxiliary variable denoting the inter-vehicle distance, $E_{d}\left[\cdot \right]$ is the average operator over the range of inter-vehicular distances, $C_{FSL}$ is the capacity of the FSL link without ground reflections, and $\tilde{C}=C/C_{FSL}$.

The expression is also the average over the inter-vehicular distance range of the cumulative distribution function (CDF) of the instantaneous capacities of the STBC with respect to the FSL solution. The outage probability in (17) provides us with the performance of the link by measuring the degradation of the signal below the threshold ξ, averaged over a given range of distances. However, the analysis of this first-order statistical metric could hide some of the dynamics in the performance of the proposed multiple antenna systems, particularly when they suffer fading or nulls. To address this issue, let us now define the *average gain in fading* as the average gain of the space-time diversity algorithm when the reference solution is in outage:

(18) $$G=E_{d}\left[C_{STBC}/C_{FSL}|\tilde{C}\leq \xi \right]=\frac{\int_{d_{min}}^{d_{max}}\left\{C_{STBC}/C_{FSL}\right\}f_{\left\{C_{STBC}/C|\tilde{C}\leq \xi \right\}}\left(x\right)d_{x}}{d_{max}-d_{min}}.$$

For Rice fading links, the outage probability of the SINR is defined as follows:

(19) $$\Theta_{\Gamma }=E_{d}\left[\mathrm{Pr}\left\{\Gamma /\Gamma_{FSL}\leq \delta \right\}\right]=\frac{\int_{d_{min}}^{d_{max}}\mathrm{Pr}\left\{\Gamma /\Gamma_{FSL}\left(x\right)\leq \delta \right\}dx}{d_{max}-d_{min}},$$

where δ is a threshold that defines the outage of the metric. A value of the metric below this threshold is assumed to be an outage event.

## 6. Evaluation

### 6.1. Simulation Setup

Consider a 2-vehicle configuration with a variable number of antennas *N* and a range of inter-vehicular distances d∈[1,20] m ($d_{min}=1$ m and $d_{max}=20$ m). The antennas are vertically polarized and distributed in two uniform linear arrays with an equal number of antennas each, i.e., $N_{Tx}=N_{Rx}=2N$. One uniform linear array is considered to be on the roof of the vehicle and the other array on the front part of the vehicle. The arrays are parallel on the y-axis and positioned at two different heights w.r.t. the ground plane, $z_{1}=1.2$ m and $z_{2}=0.6$ m. Both arrays are also separated by 1 m on the x-axis (i.e., shifted towards the front/back of the following/lead car, as depicted in Figure 1). The width of the cars is set to 2 m, over which the positions of the antennas in the array are regularly spaced according to *N*. As a feasibility constraint, we assume N≤12, and for the rest of parameters, we assume λ=0.125, $\epsilon_{r}=4$, and σ=0.02.

For the time-variant simulations, we consider two vehicle speeds of 20 km/h and 200 km/h, and two different values of Rice factor, K=−30 dB and K=15 dB. The rationale behind this selection is to test two values of Doppler frequency, which depends directly on the vehicle speed. We test a low value where Doppler shift potentially allows STBC to perform almost in the LOS regime, and another value at high speeds to test the scenario with a considerable Doppler frequency and therefore considerable time channel variations. As demonstrated in previous sections, the interference term created by the loss of orthogonality in time-varying channels can be directly or almost directly linked to the statistical deviation of the temporal components of each channel link. In all simulations, it is assumed a transmit SNR of 10 dB, which is a value relevant for the calculation of the interference term.

### 6.2. Simulation Results: LOS

**Figure 4:**
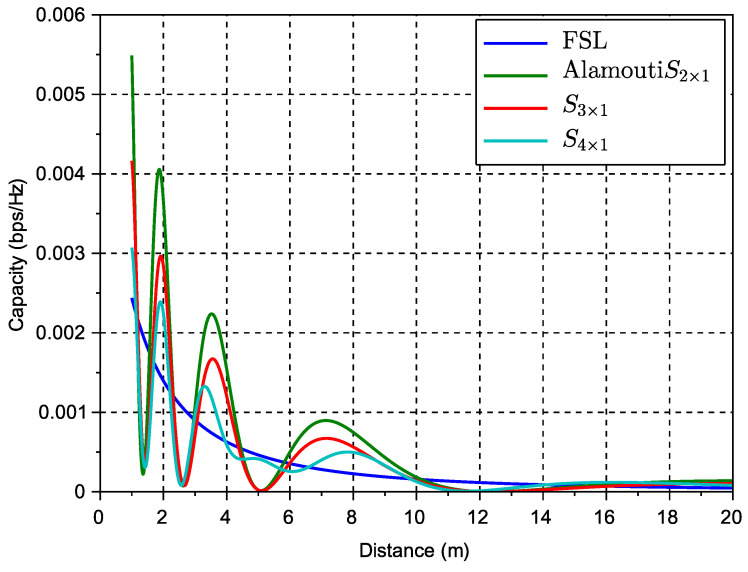
Capacity performance versus inter-vehicular distance for different STBC algorithms and the FSL solution for reference.

Figure 4 shows the capacity performance versus inter-vehicular distance for different STBC algorithms and the FSL solution for reference. Unitary power transmission was assumed.

**Figure 5:**
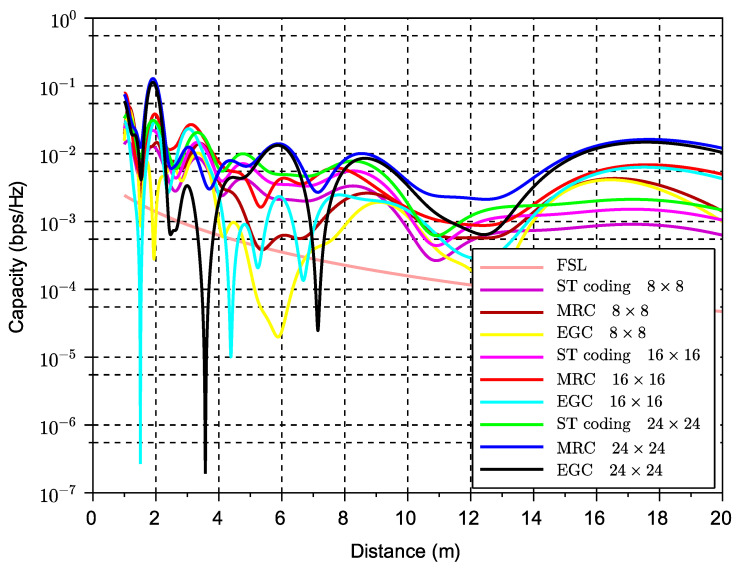
Capacity versus inter-vehicular distance of different STBC over LOS channels with ground reflection when compared to the MRC and EGC solutions ($N_{Rx}=N_{Tx}$).

Note that the results in Figure 4 consider only one antenna at the receiver (i.e., $N_{Rx}=1$). From this, we can observe a slight improvement when we use higher-order codes despite the rate loss characteristic of this type of code. The improvement is in terms of a reduction of the span between the minimum and maximum achievable instantaneous capacity. This can be seen, for example, in code $S_{4\times 1}$, which shows fewer peaks and fades than lower-order codes. Figure 5 shows capacity performance results similar to the previous case but now for higher numbers of antennas. Unitary power transmission was also assumed.

All solutions consider an abstraction of STBC schemes based on the SINR formulation in previous sections and in previous works (e.g., [11]) and the theoretical rate limit for that coding order as shown in Equation (14). The labels in all figures refer to the different schemes with the number of Tx versus the number of Rx antennas. For example, the label MRC 8 × 8 refers to the maximum ratio combining solution with 8 antennas for transmission (using symbol repetition) and 8 antennas for reception using Equation (15). Equal gain combining with 16 antennas in both ends is denoted by EGC 16 × 16 using Equation (16). Similarly, the space-time code solution with different numbers of Tx antennas and Rx antennas is denoted by ST coding $N_{Tx}\times N_{Rx}$ using Equation (11). All the schemes are compared with the free space loss, which is denoted by FSL. We recall that the coding schemes refer to an approximation of the SINR formula based on an extension of the Alamouti code for 2 × 1 and 2 × 2 STBC systems as derived in previous sections. For a more detailed description of all these solutions, including the signal model of each one as well as the concept of signal repetition for the different combining mechanisms, we refer to our previous works in [11].

Since the idea is to improve the relatively low gain obtained with only one receive antenna, we explore here the case of maximum space diversity (i.e., $N_{Rx}=N_{Tx}$). Moreover, we compare STBC performance with equivalent exclusive receive diversity schemes, particularly the MRC and EGC algorithms. Interestingly, these results show that MRC and EGC show lower and higher peaks than STBC.

**Figure 6:**
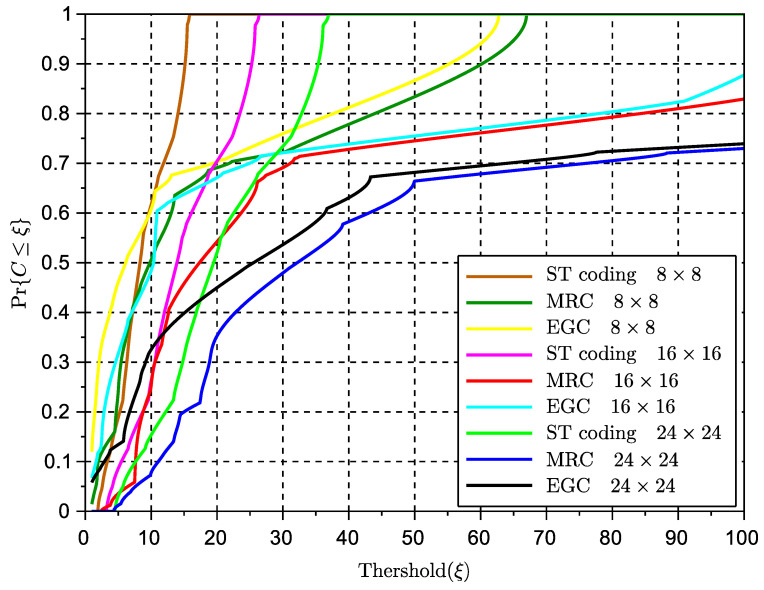
CDF of the capacity ratio of different STBCs and SIMO solutions to the capacity of the FSL case.

Figure 6 presents the cumulative density function (CDF) of the capacity performance of STBC in comparison with conventional multiple-antenna-combining solutions with symbol repetition across the transmit antennas (i.e., MRC and EGC). The formula used for this outage probability is given in the previous subsection in (17) and is calculated using well-known numerical methods for integration. The results show that STBC seems to behave worse than MRC and EGC. In addition, MRC outperforms EGC, an observation which contradicts the results in our previous paper in [11], where EGC shows a higher signal excursion than MRC. The explanation lies in the omission of the accumulation of noise in the performance of EGC in our previous paper [11], which leads EGC to show higher signal excursions than MRC.

In this paper, it is shown that with a correct formulation of the noise component of EGC, MRC seems to outperform both EGC and STBC. However, EGC still shows larger signal excursions with more pronounced fades, as found in the original paper. Note that the results of our previous paper [11] still apply to the behavior of the signal strength (ignoring the noise component). Despite these advances, the average results tend to hide some specific aspects of the performance of the solutions. In fact, our prior work also shows that EGC and MRC can be the worst solutions in terms of the depth of the fades at given values of inter-vehicular distance. That is to say, while EGC or MRC can experience the largest signal values, they can also show the deepest fades or lowest values of signal strength and also SNR.

**Figure 7:**
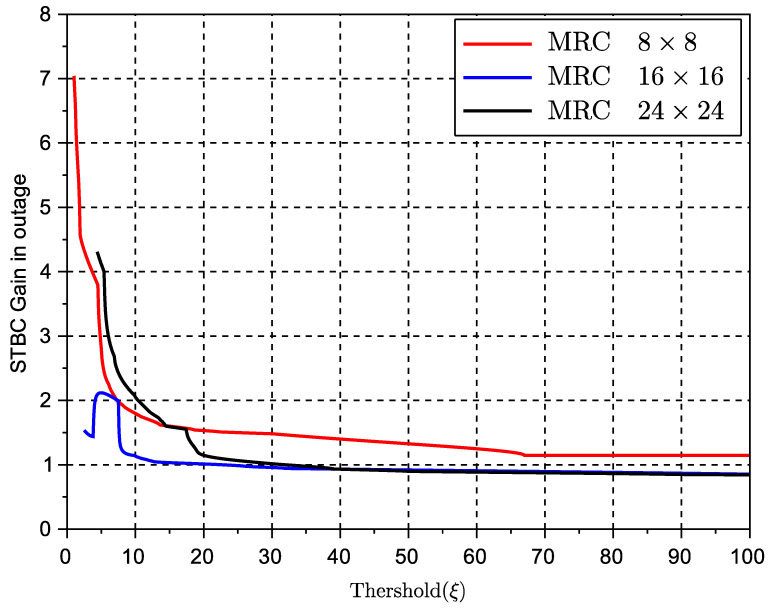
Capacity gain (G) in outage of STBCs over MRC multiple antenna schemes.

**Figure 8:**
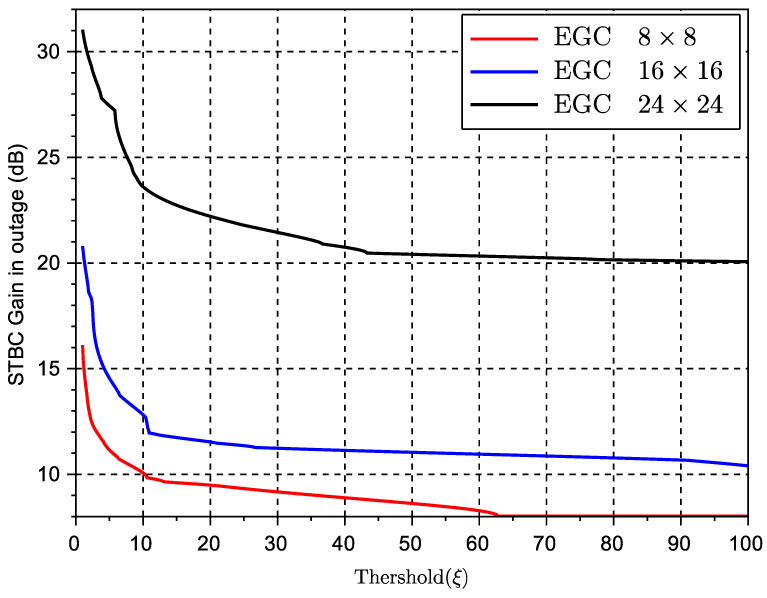
Capacity gain (G) in outage of STBCs over EGC multiple antenna schemes.

To better investigate the above-mentioned behavior, we study the metric *average gain in outage* (or average gain in fading) as defined in (18) in the previous subsection. Specifically, Figure 7 and Figure 8 show the performance of this gain with regards to the MRC and EGC solutions, respectively, for various values of the threshold used in the definition of outage probability in (17). The threshold measures the level at which a signal or metric level is considered as an outage. Obviously, the lower the threshold, the deeper the potential signal or capacity fade. Note that for better visualization, the observed gains over the MRC solution are in linear scale and the gains over EGC are in dB. These results clearly show that STBC outperforms both types of combining SIMO solutions when these solutions are in an outage stage. From this result, we can therefore conclude that while space-time diversity can limit the maximum peak of the received signal, it provides a very good countermeasure against fading or signal nulls for various ranges of inter-vehicular distance.

### 6.3. Simulation Results Time-Variant Rice Fading Links

**Figure 9:**
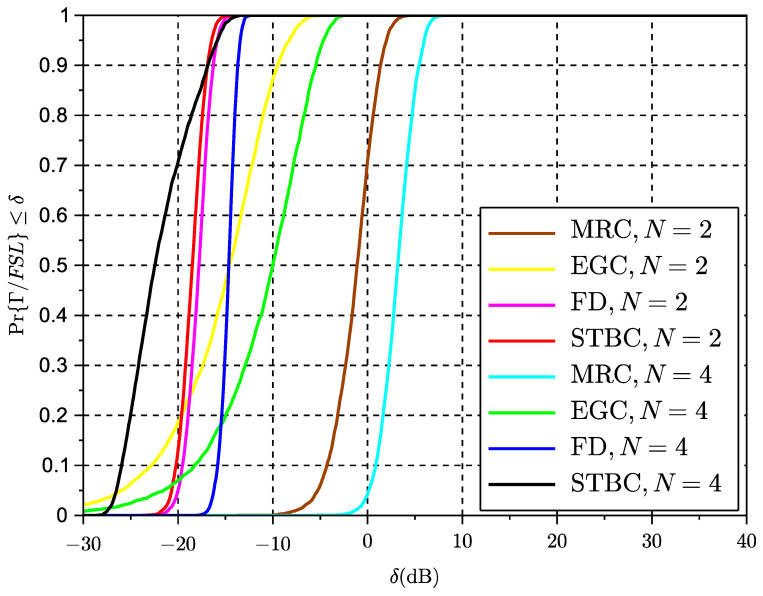
CDF of SINR for different multiple antenna schemes, K=−30 dB and v=20 km/h.

**Figure 10:**
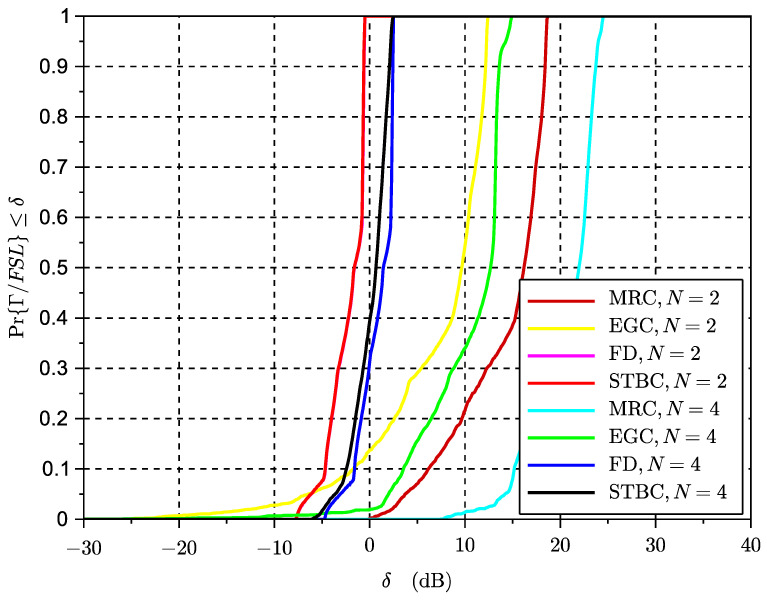
CDF of SINR for different multiple antenna schemes, K=15 dB and v=20 km/h.

**Figure 11:**
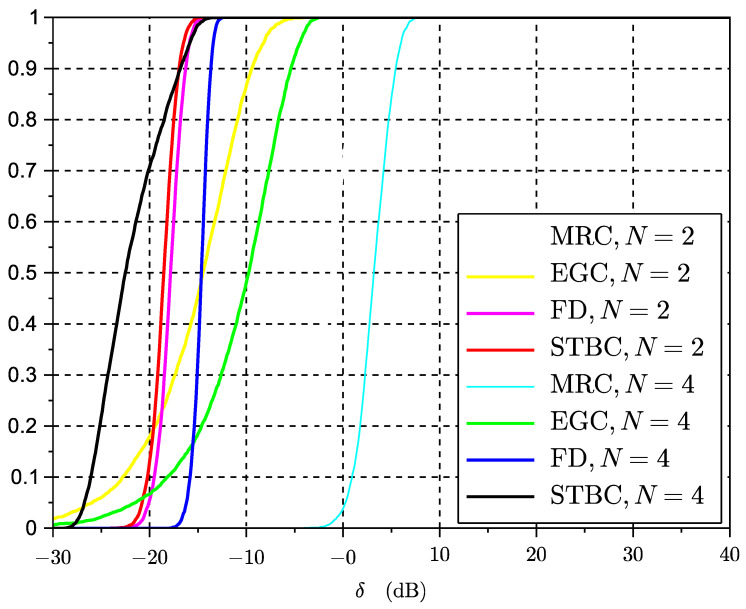
CDF of SINR for different multiple antenna schemes, K=−30 dB and v=200 km/h.

**Figure 12:**
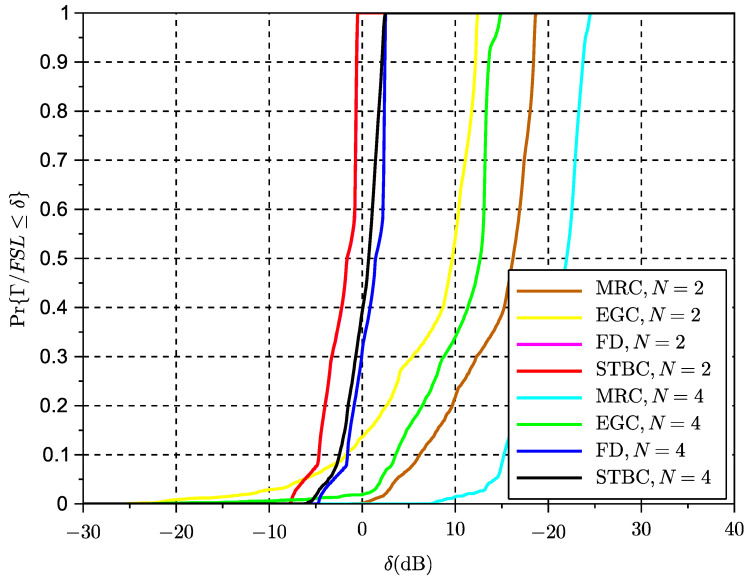
CDF of SINR for different multiple antenna schemes, K=15 dB and v=200 km/h.

The results of the cumulative distribution function of the SINR for time-variant systems are shown in Figure 9 for a Rice factor of −30 dB and in Figure 10 for a Rice factor equal to 15 dB, while the results in Figure 10 are based on a Rice factor of −30 dB. The speed of the vehicle was set to 20 km/h. In these figures, the value of the x-axis denotes the threshold δ as defined in the expression (19) for the outage of the SINR metric. The threshold is the actual value of the SINR ratio metric below which the signal is considered to be in outage. The effects of interference can be clearly observed in the results for STBC as compared to the results with exclusive receive diversity. The results show that despite the losses seeming significant in the case of reduced LOS, in some cases a higher number of antennas can reduce the interference. However, an increase in the number of antennas can also lead to an increase in the length of the space-time code and thus to the loss of initial spatial diversity gains due to an excess of temporal interference. This suggests a trade-off between the length of the code to control the interference and the possible use of additional or complementary receive diversity to increase SNR and thus reduce interference. The same exercise of simulation has been repeated for a scenario with a vehicle speed of 200 km/h in Figure 11 and Figure 12. These results seem to confirm that increased Doppler frequency can affect the orthogonality of STBC codes, but they can accept some margin of increased Doppler while providing some acceptable performance. This means these types of codes could be attractive for low-speed scenarios, which are typical of dense urban scenarios with traffic congestion or limited speed. On the other hand, we confirm that schemes with exclusively receive diversity are unaffected by the emulated channel time variations. The main conclusion from the analysis in LOS and NLOS conditions is that STBCs mainly provide gain over the rest of the solutions when there is a deep fade and also when the Doppler shift is not significant, so the degradation due to loss of orthogonality does not surpass the gain in outage due to the reversal of the deep fade thanks to spatial diversity. This analysis opens the door to a new type of STBCs that can show an interesting trade-off between the loss of orthogonality in time-varying channels, their fading rejection capability, and the length or rate of the block code. It is an interesting future work to investigate a wider range of network coding schemes for MIMO, verify the general findings observed in this paper regarding the variables in trade-off, and design new or select existing coding strategies that better suit a given channel profile.

IES

## 7. Conclusions

This paper has presented the performance analysis of orthogonal space-time block codes in V2V MIMO channels with two particular issues: ground reflections that affect the LOS component and time-variant channels that destroy the code orthogonality. These two impairments are expected to appear in scenarios with higher vehicle speed, reduced inter-vehicular distances, and in dense urban environments with rich scattering distributions. The results show that STBCs can suffer severe degradation in high-Doppler scenarios (higher vehicular speeds, higher operational frequency, or shorter transmission periods), but this effect can be reduced by an increase of the Rice factor and also an increase in the number of antennas used over the vehicles. In the pure LOS regime, STBCs can provide capacity gains over single-antenna systems as well as reduce the effects of self-interference produced by ground reflections. Moreover, when compared to conventional SIMO solutions using EGC and MRC schemes, STBC seems to perform rather poorly (on average) regarding outage probability for different values of performance ratio with respect to the FSL solution. However, a closer look at the performance of the solutions in the case of outage for high Rice factors reveals that STBC provides gains in performance with respect to MRC and, particularly, with respect to EGC when these solutions are in outage. This implies that STBC is an attractive candidate to counteract deep fades and provide a more stable signal level (here against self-interference patterns), but at the same time, it cannot provide the maximum signal peaks as compared to MRC or EGC. Another conclusion is that despite losses in code rates, STBC in combination with multiple antenna receivers can provide important capacity gains for the LOS components when affected by ground reflections.

Higher-order STBCs provide a higher degree of rejection to deep fades. However, in time-variant channels, their performance degrades faster. Therefore, the conclusion is to use low-order STBCs in fast-fading channels. Our results aim to help in the design and optimization of the vehicular networks of the future by predicting the behavior of different types of MIMO solution and under which circumstances they provide desired gains.

Future work includes the validation on experimental testbeds for STBCs in V2V MIMO and the experimentation of STBCs.

## Data Availability

Data are contained within the article.

## Abbreviations

The following abbreviations are used in this manuscript:

STBC	Space-Time Block Coding
MRC	Maximum Ratio Combining
EGC	Equal Gain Combining
MIMO	Multiple-Input Multiple-Output
V2V	Vehicle-to-Vehicle
LOS	Line-of-Sight
SNR	Signal-to-Noise Ratio
SINR	Signal-to-Interference-plus-Noise Ratio
CDF	Cumulative Distribution Function
NLOS	Non-Line-Of-Sight
FSL	Free Space Loss
SIMO	Single-Input Multiple-Output
MISO	Multiple-Input Single-Output
